# The influence of associative reward learning on motor inhibition

**DOI:** 10.1007/s00426-021-01485-7

**Published:** 2021-02-17

**Authors:** Janina Rebecca Marchner, Claudia Preuschhof

**Affiliations:** 1 Department of Clinical Developmental Psychology, Institute of Psychology, Faculty of Natural Sciences, Otto-von-Guericke-University, Universitätsplatz 2, Gebäude 24, 39106 Magdeburg, Germany; 2 Center for Behavioral Brain Sciences, 39106 Magdeburg, Germany

**Keywords:** Attentional capture, Associative learning, Reward, Inhibition, Cognitive control

## Abstract

Stimuli that predict a rewarding outcome can cause difficulties to inhibit unfavourable behaviour. Research suggests that this is also the case for stimuli with a history of reward extending these effects on action control to situations, where reward is no longer accessible. We expand this line of research by investigating if previously reward-predictive stimuli promote behavioural activation and impair motor inhibition in a second unrelated task. In two experiments participants were trained to associate colours with a monetary reward or neutral feedback. Afterwards participants performed a cued go/no-go task, where cues appeared in the colours previously associated with feedback during training. In both experiments training resulted in faster responses in rewarded trials providing evidence of a value-driven response bias as long as reward was accessible. However, stimuli with a history of reward did not interfere with goal-directed action and inhibition in a subsequent task after removal of the reward incentives. While the first experiment was not conclusive regarding an impact of reward-associated cues on response inhibition, the second experiment, validated by Bayesian statistics, clearly questioned an effect of reward history on inhibitory control. This stands in contrast to earlier findings suggesting that the effect of reward history on subsequent action control is not as consistent as previously assumed. Our results show that participants are able to overcome influences from Pavlovian learning in a simple inhibition task. We discuss our findings with respect to features of the experimental design which may help or complicate overcoming behavioural biases induced by reward history.

**Supplementary Information:**

The online version contains supplementary material available at 10.1007/s00426-021-01485-7.

## Introduction

Our experience with reward robustly influences what we pay attention to in future situations and our eyes are immediately drawn towards aspects in the visual field that were previously associated with a rewarding outcome (Anderson, 2015; Anderson & Halpern, 2017; Camara, Manohar, & Husain 2013; Failing & Theeuwes, 2015, 2017; Failing, Nissens, Pearson, Le Pelley, & Theeuwes, 2015; Le Pelley, Pearson, Griffiths, & Beesley, 2015; Marchner & Preuschhof, 2018; Mine & Saiki, 2015; Pearson, Donkin, Tran, Most, & Le Pelley, 2015; Pool, Brosch, Delplanque, & Sander 2014). The ‘history of reward’ guides visual attention in a rather automatic fashion (Theeuwes, 2018), which is advantageous as long as it is in line with our current goals and intentions. But we also preferentially attend reward-associated stimuli under conditions, where it is no longer helpful or even entails negative consequences (e.g.,Camara et al., 2013; Failing & Theeuwes, 2017; Le Pelley et al., 2015). The learning of stimulus–response–outcome associations does not only lead to behavioural changes but also leads to neural changes in the visual cortex reflecting habit-like prioritisation of value-signalling stimuli at an early processing stage (Luque et al., 2017). We are, therefore, not completely flexible to choose what we attend to in a controlled manner. Instead, modulations of attentional processes through associative reward learning can impede voluntary cognitive control (Failing & Theeuwes, 2017; Le Pelley et al., 2015) and may also contribute to undesirable behavioural choices and actions (Anderson, 2017; Camara et al., 2013). The mechanism by which reward history is translated into behaviour is not clear and remains an interesting research field for further investigations.

Some studies suggest that reward-associated but irrelevant stimuli facilitate action towards these stimuli despite contrary intentions (see Anderson, 2017 for a review). Stimuli with a history of reward can impact cognitive control, which enables planning and execution of advantageous behaviour as well as the ability to manage conflicting information and inhibit undesirable responses in accordance with internal goals and intentions (Bühringer, Wittchen, Gottlebe, Kufeld, & Goschke, 2008; Cole & Schneider, 2007; Miller & Cohen, 2001; Monsell & Driver, 2000). For example, when participants are asked to make choices upon two options with differing reward potential, their decisions are slowed and less optimal when a task-irrelevant but reward-associated distractor is present (Gluth, Spektor, & Rieskamp, 2018; Itthipuripat, Cha, Rangsipat, & Serences, 2015). It was argued that reward-associated stimuli capture attention, causing interference, thereby leading to the decline in performance during decision-making.

Interference effects by task-irrelevant but reward-signalling stimuli were also observed in a Stroop task (Liao, Grégoire, & Anderson, 2020; Krebs, Boehler, Egner, & Woldorff, 2011, 2013). In one study neural activity in a prefrontal motor-control area was increased by irrelevant reward-predictive stimuli, which could reflect cognitive control processes to overcome unfavourable response tendencies that are automatically triggered when individuals encounter a stimulus related to reward (Krebs et al., 2011). Also, when flankers conflict not only with the signal to go for a response, but are also associated with value, they interfere with otherwise typically found inhibitory processes altering response speed (Anderson et al., 2016; Kim & Anderson, 2019). Furthermore, inhibition of impulsive responses was modulated by previously reward-predictive stimuli in another study using a variant of the Simon task (Wouwe, van den Wildenberg, Ridderinkhof, Claassen, Neimat, & Wylie, 2015), a task that measures manual responses to spatially congruent or incongruent visual targets.

In summary, the power of previously reward-predictive features to automatically attract attention and hinder voluntary cognitive control is supported by studies examining the influence of reward history on both attentional selection and on choices and conflict resolution. One interpretation is that encountering a previously reward-predictive stimulus automatically generates approach tendencies, because responding towards these features was advantageous in the past (Anderson et al., 2016) and that the generated impulse to approach subsequently impairs goal-directed behaviour, when approaching is not functional. Research from animal and human studies suggests the existence of ‘natural’ Pavlovian biases in which valence and action are coupled—reward promoting activation/approach and the prospect of punishment facilitating withdrawal/inhibition (e.g., Hershberger, 1986; Huys, Cools, Gölzer, Friedel, & Heinz, 2011; Guitart-Masip, Huys, Fuentemilla, Dayan, Duzel, & Dolan 2012; for a review see Guitart-Masip, Duzel, Dolan, & Dayan, 2014). On the other hand, the prospect of reward can also improve inhibitory performance, especially when participants are informed about the reward magnitude and with higher reward feedback at the beginning of the task (Boehler, Hopf, Stoppel, & Krebs, 2012, 2014; Herrera, Speranza, Hampshire, & Bekinschtein, 2014, Herrera, Van Meerbeke, Speranza, Cabra, Bonilla, Canu, & Bekinschtein,^2019^). When reward-associated stimuli are rendered irrelevant for the current task, inhibitory control seems to be disrupted by both congruent action-valence couplings (action-reward, and inaction-punishment) but not by conflicting ones (inaction-reward, action-punishment; van Wouwe et al., 2015). As inhibitory control was modulated by the presence or absence of a response conflict rather than by the outcome valence (van Wouwe et al., 2015), an alternative interpretation may be that inhibitory processes are only triggered in the case of a valence-action conflict, while they may be disengaged when processing congruent valence-response couplings (Cavanagh, Eisenberg, Guitart-Masip, Huys, & Frank, 2013; Guitart-Masip et al., 2012; van Wouwe et al., 2015). From this perspective reward-history might affect attention and behavioral control, because the natural action-valence couplings disengage inhibitory processes, thus making it harder to inhibit unfavorable actions. Cognitive control could be self-regulated through associative learning in such a way that stimuli do not only become linked to a certain outcome and are, therefore, more or less appetitive, but also become associated with control demands/settings during learning (Abrahamse, Braem, Notebaert, & Verguts, 2016). In congruent valence-action couplings control demands may be lower which could deactivate inhibitory processes and impede inhibitory control during subsequent information processing.

Research is currently trying to disentangle whether the reported findings are caused by a direct activation of motor areas that is induced by the appetitive signal of reward-associated stimuli or whether these stimuli hinder cognitive control processes in some other way. In a recent study measuring functional magnetic resonance imaging (fMRI) and electroencephalography (EEG) using a Simon task it was investigated how reward-seeking and cognitive control are coordinated in the brain (Wang, Chang, Krebs, Boehler, Theeuwes, & Zhou, 2018). The automatic tendency to respond with the target-congruent hand was potentiated by reward and related to activation of the motor cortex. Inhibition of this process to overcome inappropriate activation was mediated by activity in the inferior frontal cortex. The authors emphasised that this pattern fits well with a 2-stage model of response activation and inhibition (Freeman & Aron, 2016; Freeman, Razhas, & Aron, 2014) proposing that reward-predictive stimuli automatically activate the motor cortex during the first stage, while during the second stage motor activation is inhibited in a controlled manner when the activated response does not match current intentions. This model also fits with a recent fMRI study, where suppression of reward-related activity in the motor cortex was found to be more effective with lower value (Kim & Anderson, 2019). Thus, first evidence is provided for a direct influence of reward-associated stimuli on motor cortex responsiveness (Kim & Anderson, 2019; Krebs et al., 2011; Wang et al., 2018), which requires the recruitment of inhibitory processes if responses are inadequate (Freeman & Aron, 2016; Wang et al., 2018).

**Fig. 1:**
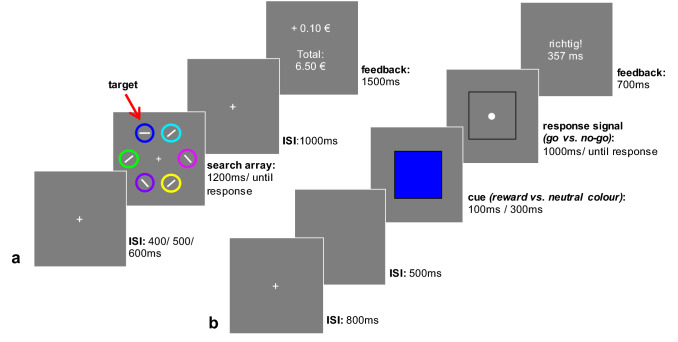
Sequence of events of the training phase (**a**) and the test phase (**b**) of experiment 1a. **a** Participants searched for a dark blue or orange coloured circle surrounding a horizontal or vertical target line and reported its orientation. Correct answers were followed by a reward (10 Cents) or a neutral feedback (“e t0 C + n0”). The reward delivery was probabilistic (20% neutral vs. 80% reward feedback). **b** In the test phase participants performed a cued go/no-go task. Subjects responded to a white circle with a button press and inhibited the response when a white diamond signalled a no-go trial. For half of the participants (go-cue: reward colour) a cue in the previously rewarded colour was followed by a go signal in 80% of the trials and in 20% by a no-go signal. In the other half of the sample (go-cue: reward colour) the same pattern of response signals was displayed following the neutral colour-cue. Stimulus onset intervals of the cue varied between 100 and 300 ms evenly distributed over trials and conditions

In the current study we aimed to extend the relatively small line of research using 2-phasic designs including a learning phase and a later test phase to investigate if previously reward-predictive features induce disinhibition when the reward signal conflicts with task goals (Anderson et al., 2016; Kim & Anderson, 2019; van Wouwe et al., 2015; Liao et al., 2020). Biases of action selection induced by reward-associated stimuli have often been studied under conditions, where reward is still available (Itthipuripat et al., 2015; Gluth et al., 2018; Krebs et al., 2011, 2013; Wang et al., 2018; Freeman & Aron, 2016) or using the same or a very similar task for the learning and test phase (e.g.,Huys et al., 2011; Freeman et al., 2014). The first aspect is important, because the reward incentives are well known to motivate goal-directed behaviour. When rewards are still available cognitive processes linked to the prospect of reward cannot be distinguished from goal-directed processes in the presence of incentives with a history of reward (Anderson and Sali, 2016). Second, from studies using addiction-related stimuli in vulnerable populations it can be inferred that valanced stimuli disrupt inhibitory control (Pike, Marks, Stoops, & Rush, 2015; Weafer & Fillmore, 2012, 2015), but studies on Pavlovian conditioning in healthy populations, which control the Pavlovian conditioning experimentally, typically use the same task for both the learning and the later test phase and sometimes rewards are continuously delivered in the test phase to ensure motivation and prevent extinction (e.g., Huys et al., 2011; Freeman et al., 2014; also see Cartoni et al., 2016 for a review). We were interested in the potential for generalization of value-driven effects beyond a specific learning context towards other domains, impacting response inhibition in situations, where the reward signal is completely irrelevant. For this purpose we combined the training phase of a typical value-driven attentional capture task with an unrelated cued go/no-go task (Weafer & Fillmore, 2012; attentional-bias behavioural activation—ABBA task; see Fig. 1) in two experiments. To our knowledge, this is the first study using a go/no-go task in test phase to probe the effect of reward history on motor inhibition. In the training phase participants experienced certain colours in close spatio-temporal proximity to a rewarding outcome, which typically biases attentional selection. We expected that associative learning would improve performance in the training phase but induce more failures to suppress undesirable responses in the subsequent cued go/no-go task, because the cue colours were linked to valuable outcome in the training phase. This finding would contribute to the growing evidence that incidentally learned contextual reward information later biases processes of behavioural activation and inhibition in favour of irrelevant, but value-signalling stimuli, handicapping controlled responding.

## Experiment 1

### Materials and methods

*Participants* In total 77 university students took part in the experiment. One subject was excluded because of low performance (below 70% correct responses) in the training phase. Data of 76 participants (mean age: 23.20 years, age-range: 18–33 years; 52 females and 24 males) were included into the statistical analyses. Participants had no record of psychiatric or neurological illness (including abuse of nicotine, alcohol, drug or medication) and a normal BDI-II score (sum < 14; Beck depression inventory; Hautzinger, Keller, & Kühner, 2006) on the day of measurement. Colour vision (Ishihara, 2010) was tested and visual acuity was (corrected to) normal. Participants were naïve to the purpose of the study. A post-experimental questionnaire (following Anderson et al., 2011) revealed that 33 (versus 43) participants recognized the correct colour-feedback association with high or low certainty.

*Apparatus* Computer-based tasks were programmed and run using Matlab 2012b (The MathWorks, Inc., 2012) and the Psychophysics Toolbox 3.0.12 (Brainard, 1997; Kleiner et al., 2007; Pelli, 1997). Stimuli were displayed on a Samsung S24C450 monitor (24″, TN panel, 1920 × 1080 pixel, 60 Hz refresh rate) positioned on a desk. Participants were seated in an office chair behind a desk in separated experimental cabins painted in black. Viewing distance was approximately 60 cm. Responses were given on a standard keyboard.

*General procedure* The entire experimental session lasted about two hours. After giving written informed consent participants completed a test for colour deficiency (Ishihara, 2010) and a screening for acute depressive symptoms (Beck depression inventory II, by Hautzinger et al., 2006). Afterwards the training phase of the experiment was explained and practiced for 30 trials directly prior to its start. Participants were informed that they could win a maximum of 9.60 Euros during the training phase of the experiment and that half of this amount would be paid out in addition to the compensation of 14 € right after the experiment. Subsequently participants were instructed for the ABBA task and then performed the test phase of the experiment.

*Training Value-Driven Attentional Capture (VDAC)* A modified version of Anderson et al.'s (2011) paradigm was used to incidentally train stimulus-reward associations. The main modification involved contrasting reward feedback with a ‘neutral’, none-monetary feedback, instead of lower reward feedback. This modification was performed, because we were interested in effects driven by value and it has been shown that when a stimulus repeatedly appears together with the target during visual search it can also bias responses in subsequent tasks (e.g.,Anderson et al., 2017; Sha & Jiang, 2016; Wang et al., 2013). The chosen design has proven to be efficient in a previous study about attentional selection processes, where training effects generalized to a subsequent visual search task (Marchner & Preuschhof, 2018). We also decided not to use red and green as target colours, because they are associated with going and stopping in traffic, which could impact measurements in a go/no-go paradigm. Figure 1a displays the sequence and timing of the training phase. All stimuli were presented against a grey background. Each trial started with the presentation of a white fixation cross, which lasted for either 400, 500, or 600 ms. Then an array composed of six coloured circles (orange, blue, green, magenta, cyan, orange, yellow, lilac or white—each 2.58° × 2.58° visual angle) placed on an imaginary circle with a radius of 5° was presented. Inside each circle a white line was shown. The target stimulus was a vertically or horizontally oriented line which was surrounded by a coloured circle (blue or orange) associated with one of the experimental conditions (reward or neutral) via feedback. The other lines were randomly presented at different angles (rotation increments of 45°). Participants were instructed to search for a white line in orange and blue circles and report its orientation as fast as possible by either pressing the left arrow key for vertical lines or the right arrow key for horizontal lines. The stimulus array was presented until a response was given or until a maximum response time of 1200 ms was reached. Subsequently the white fixation cross appeared for another 1000 ms. Then feedback was given for 1500 ms. The feedback used to incidentally establish associations with colour, was either monetary (10 Eurocents; “ + 10 Cents”) or ‘neutral’ (of no monetary value). Additionally, the sum of the total money gained was presented on screen. The feedback presented during neutral trials consisted of a nonsense string of letters used for the reward feedback (“mst:meGmsuae rEou “). Answers that were incorrect or too slow were followed by a feedback indicating an error (“Fehler”—the German word for error). To reduce the likelihood that the subjects realised the colour-reward associations, reward delivery was probabilistic during rewarded trials (20% neutral vs. 80% reward feedback), while the neutral colour was never followed by a reward feedback. The training phase consisted of 40 trials per run and per experimental condition (reward and neutral feedback) resulting in a number of 240 total trials. A break of 30 s separated the three experimental blocks. Conditions as well as orientation and location of the target stimulus were evenly distributed over trials and colours were counterbalanced across participants to control for potential differences in perceptual salience. Trials were presented in a pseudo-randomized fashion per block so that the same experimental condition and type of target did not appear successively for more than three trials.

*Testing attentional-bias behavioural-activation (ABBA)* In the test phase participants performed a cued go/no-go task originally developed to measure if in addicted populations a drug-related attentional bias disrupts behavioural control (Weafer & Fillmore, 2012). In our experiment cues were not addiction-related but previously reward-predictive or associated with non-monetary feedback. Figure 1b depicts the sequence and time course of the test phase. Each trial started with a white fixation-cross presented in the centre of the screen for 800 ms followed by an empty screen (inter stimulus interval; ISI) presented for 500 ms. Then a square (12.90° × 12.90° visual angle) was presented in one of the colours (blue, orange) that was associated with a monetary or a neutral feedback during the training phase. The cue lasted 100 ms or 300 ms resulting in two stimulus-onset asynchronies (SOA) to allow the examination of the temporal dependency of the expected effects. Afterwards the colour cue disappeared, the black outline of the square remained on the screen and a response signal was displayed centrally within the square. In go-trials participants were instructed to respond to a white circle (0.52° × 0.52° visual angle) by pressing the left arrow key. In no-go trials a white diamond (0.52° × 0.52° visual angle) appeared and participants had to withhold their response. The response screen lasted until a response was given or ended after a maximum response time of 1000 ms. Participants were informed that they would not be rewarded in this task but that a feedback would indicate the accuracy of their responses. Performance feedback was given for 700 ms using the German words for correct and incorrect (“richtig!”; “falsch!”) and the response time was shown in correctly answered go-trials.

The ABBA task was performed over 7 blocks of 40 trials resulting in a total number of 280 trials. The influence of value was investigated in a between-subject design: In the go-for-reward group (go-cue linked to reward colour) the previously rewarded colour cue was followed by a go signal in 80% of the trials and by a no-go signal in 20% of the trials (112 versus 28 trials), whereas the cue presented in the neutral colour was followed by a go signal in 20% of the trials and by no-go signal in 80% of the trials. In the go-for-neutral group (go-cue linked to neutral colour) the pattern was reversed so that the neutral colour cue predicted to go for a response, while the reward associated colour predicted to withhold a response. Cues provided two kinds of information. On the one hand cues informed about the probability of the required response in each trial. Learning these probabilities should improve performance during the course of the experiment after valid cues (when the predictive value of the cue matched the response signal) and become more and more complicated after invalid cues (when the cue signals the contrary response). On the other hand, cues were also associated with previous feedback (reward vs. neutral) which allowed to examine the effect of previously established reward associations on behavioural activation and inhibition by comparing the two experimental groups. We expected that cues associated with a reward would cause activation or promote readiness to respond in terms of facilitated approach motivation while making it harder to inhibit responses. Thus, we hypothesised participants would respond faster after valid go-cues, if their colour was previously associated with a reward and that invalid reward-associated cues would cause a higher percentage of inhibition errors in no-go trials. Half of the participants was randomly assigned to each of the two groups (38 subjects each were compared). Colour randomisation was fully counterbalanced in the total sample and within the two groups. SOA conditions were evenly distributed over trials and experimental conditions were presented in a pseudo-randomized fashion per block.

*Power calculations for experiment 1* To estimate an adequate sample size for the experiment power calculations were performed a priori using GPower 3 (Faul, Erdfelder, Lang, & Buchner, 2007). The calculations were performed setting α = 0.05 and β = 0.85 assuming a medium effect size (*f* = 0.25). We focussed on the most interesting effects, the between-group differences in a repeated analysis of variance. The analysis revealed a sufficient sample size of N = 74 participants with an actual power of β = 0.85.

*Statistical approach* Preparations for statistical analyses were performed using SPSS (IBM Corp., 2013). Bayesian statistical analyses were carried out using JASP (JASP Team, 2020, Version 0.14) with standard settings for priors. The Bayesian statistical approach determines the probability of the data to be found under the alternative hypothesis and the null hypothesis. To evaluate interaction effects, we followed the method suggested by Sebastiaan Mathôt, which is integrated into JASP as the BF-inclusion factor in effects across matched models. The method divides the sum of P(M|data) of all models including the interaction term of interest (but excluding the three-way interaction) by the sum of P(M|data) of the same models stripped by the interaction term of interest. For reporting results of the Bayesian analyses, we followed the language suggested by Lee and Wagenmakers (2013) adjusted from Jeffreys (1961). For the training phase we conducted repeated analyses of variance for accuracy and response times and for the test phase we conducted repeated analyses of variance for accuracy in no-go trials following go-cues (as a measure of response inhibition) and response time in go-trials following go-cues (as a measure of approach activation). All other results can be found in the supplementary material. The supplementary material additionally provides analyses of variance using SPSS (IBM Corp., 2013) for all task measures of the training and test phase data of experiment 1 and 2.

### Results of experiment 1

#### Training phase

**Fig. 2:**
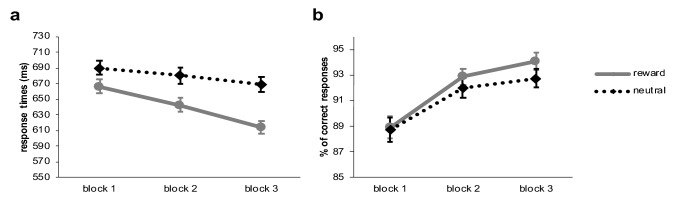
Response times of correct responses (**a**) and accuracy of responses (**b**) per block and feedback condition during training of experiment 1. Error bars reflect the standard error of means

For every subject and every experimental condition separately response time data for correct trials were cleaned by removing values three standard deviations above and below the individual mean. As a result, 0.7% of trials were excluded from further analyses. One participant responded incorrect in more than 30% of the trials and was, therefore, excluded. Accuracy in the training phase of the remaining (N = 76) participants was 91.55%. Correct response time and accuracy were then analysed using Bayesian repeated measures analysis of variance with the within subject factors feedback type (reward, neutral) and block. Figure 2 depicts response times and accuracy of responses over the time course of the training phase for the experimental conditions.

The Bayesian repeated-measures analysis of variance decisively supported the influence of feedback type on response times in the training phase (BF10 = 4.595e + 9), with responses being faster during rewarded trials. We also found extreme evidence that responses became faster over the time course of the experiment (BF10 = 28,408.249). The analysis for an interaction effect between feedback type and block was not informative with a Bayesian factor close to zero (BF-inclusion = 0.779). However, it should be noted that we observed a steeper increase of response speed in rewarded trials compared to unrewarded feedback trials which was statistically significant in the frequentist analysis (compare supplementary material).

The same Bayesian repeated-measures ANOVA was calculated for accuracy of responses. Performance improved overall throughout the time course of the training phase and this effect was extremely unlikely under the null hypothesis (BF10 = 3.397e + 10). Accuracy of responses was higher in rewarded compared to unrewarded trials, and the Bayesian factor suggested the effect was three-times more likely to be found under the assumption of the null hypothesis (BF10 = 0.307). Results for the interaction between block and feedback type revealed strong evidence for the null hypothesis, suggesting there was no difference between the conditions over time (BF-inclusion = 0.070).

#### Test phase

**Fig. 3:**
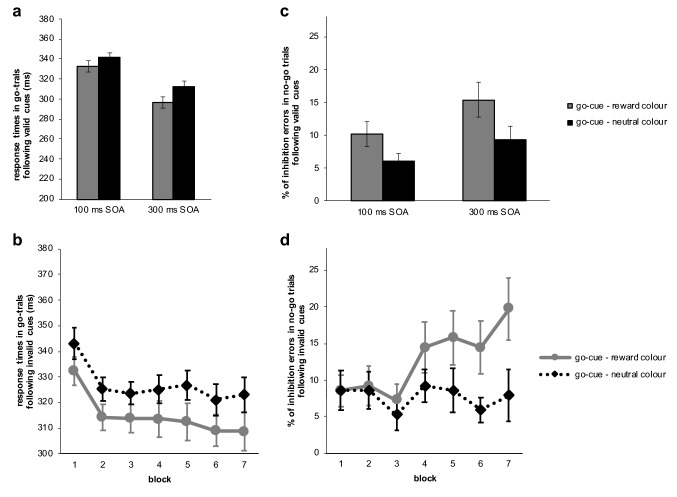
Results of the test phase of experiment 1: graph **a** shows response times in go-trials following go-cues per SOA and graph **b** per block. Graph **c** and **d** illustrate the same but for accuracy of responses in no-go trials following go-cues, which was converted into percentage of inhibition errors for illustration purposes. Error bars reflect the standard error of means

Cleaning and statistical analysis of the ABBA task data was performed using the same procedure as for the training phase data. In total 0.1% of the responses during go-trials were removed from the test phase data. Average accuracy in the test phase was 99.72% for go-trials and 95.22% for no-go trials. We conducted Bayesian repeated-measures analysis of variance with the within factors block and SOA and the between subject factor experimental group. View Fig. 3 for an illustration of the results.

*Accuracy in no-go trials following (invalid) go-cues:* When participants had to inhibit a prepotent response, errors were more frequent in the group that had associated the cue with reward compared to subjects who had previously associated the cue colour with neutral feedback (Fig. 3c, d). The frequentist analysis suggested a significant reward-associated group difference (compare supplementary material). However, the Bayesian factor was very close to zero (BF10 = 0.773), suggesting that the data was not informative about whether inhibitory performance was influenced by irrelevant reward-associated cues. Overall less inhibition errors were made when responses were given after 100 ms, while we observed more errors with a longer SOA of 300 ms (BF10 = 14.260). The Bayesian analysis spoke against an interaction effect between SOA and block (BF-inclusion = 0.016) as well as SOA and experimental group (BF-inclusion = 0.124; view also Fig. 3c). There was strong evidence against the assumption that inhibition errors overall varied with repetitions (block: BF10 = 0.055). Graphical exploration of the data implied differences in learning of the probabilities between the experimental groups (see Fig. 3d). However, the Bayesian analysis did not speak for this interpretation as the interactions between experimental group and block (BF-inclusion = 0.051) as well as three-way with SOA (BF-inclusion = 0.017) suggested the data was (very) strongly in favour of the null hypothesis.

*Response times in go-trials following (valid) go-cues:* Regarding the factor experimental group we observed a Bayesian factor around zero suggesting the data was indecisive regarding a value-driven bias of response times in this sample (BF10 = 0.818). Figure 3b shows the mean response times per group in valid go trials over time. The figure also illustrates that responses became overall faster over the course of the test phase (block: BF10 = 3.436e + 6). Unsurprisingly we found extreme evidence that response times were shorter when 300 ms compared to 100 ms elapsed between the cues and the go-signal (SOA: BF10 = 1.009e + 85) and against the assumption of an interaction between factor SOA and block (BF-inclusion = 0.008). Concerning an interaction effect of experimental group with SOA the analysis was not providing conclusive evidence (BF-inclusion = 0.147; also view Fig. 3a). Importantly, the Bayesian analysis suggested extreme evidence in favour of the null hypothesis concerning an interaction between block and experimental group (BF-inclusion = 0.002; Fig. 3b), which implies that learning of the probabilities with which cues were followed by a go-signal did not differ depending on the associations with neutral or reward feedback. There was also very strong evidence for the null hypothesis in case of the three-way interaction (BF-inclusion = 0.016).

*Discussion of experiment 1* First, statistical analysis (both, the Bayesian and frequentist approach) provided clear evidence for successful learning of stimulus–response–outcome associations throughout the training phase. The prospect of reward feedback enhanced performance expectedly. However, in contrast to findings from former research we found no convincing evidence that would indicate learned stimulus–outcome associations interfere with goal-directed response inhibition when they are irrelevant for the current task. The frequentist analysis suggested a disinhibitory effect of irrelevant stimuli with a history of reward, and the conducted Bayesian analysis was not decisive regarding the presence of a value-driven disinhibition in the test phase. Taken together we concluded that there was more data needed for an informative result.

We did not observe differences in learning of the probabilities between the experimental groups. Although graphical exploration of the data (Fig. 3d) suggested such differences in learning, all statistical analysis showed there was no interaction between experimental group and block evident in the data. However, we would like to point out that learning can be promoted by the context of reward and could cause a value-driven group difference for example through heightened attention triggered by the formerly reward predictive cues. Because in the design of the first experiment cues carried information about both—the contingencies to learn as well as the reward information—it is not possible to clearly distinguish the effects of value on learning of the contingencies from a more direct effect of value on inhibitory performance. For these reasons we decided to conduct a second experiment in which we aimed to clarify the unexpected results from the first experiment comparing the effects of reward history within-subject and also separating the value signalling colour cue from cues carrying information about the contingencies.

Overall response times were shorter and less accurate when 300 ms compared to 100 ms elapsed between cues and the response signal. Faster responses in the longer SOA are likely due to the longer processing time available. The occurrence of a higher error rate in the longer SOA is surprising and suggests that 300 ms is not enough time for more goal-directed processes to prevent impulsive behavioural responses. More importantly, SOA did not interact with experimental group, which indicates that in our sample groups did not differ with respect to the processing time available.

## Experiment 2

In a second experiment we examined the influence of reward history on response inhibition in an independent goal-directed task using a within-subject design. The task design was very similar to experiment 1, with the exception that during the test phase we included a condition (two fractal images were presented as cues) manipulating the probability of the go-response. We also omitted the SOA-manipulation. Ethical procedure, apparatus, general procedure and the training phase were identical in experiment 1 and 2.

*Participants* For experiment 2 we recruited 76 university students. Five participants were excluded from further analysis due to low performance (error rate over 30%) in the training phase, resulting in a sample of 71 subjects for statistical analyses (mean age: 22.18 years, age-range: 18–34 years; 56 females and 15 males). Feedback colours were nearly fully counterbalanced between conditions (36 subjects learned to associate blue with reward and orange with neutral feedback; 35 subjects were trained to the opposite).

**Fig. 4:**
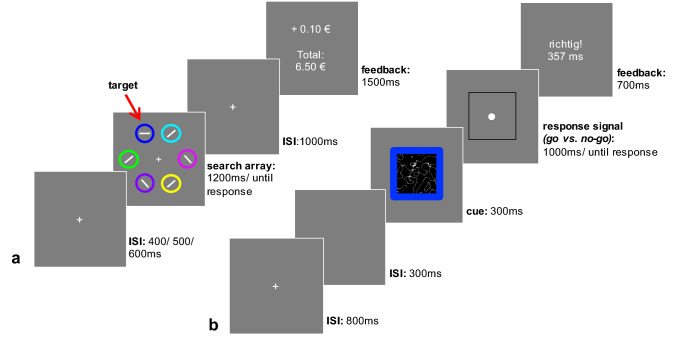
Sequence of events of the training phase (**a**) and the test phase (**b**) of experiment 2. **a** Participants searched for a horizontal or vertical target line colored in white and reported its orientation. The target line was either surrounded by a dark blue or an orange colored circle, which participants learned to associate with reward (10 Cents) or neutral feedback (“e t0 C + n0”) over the course of the training phase. **b** In the subsequent cued go/no-go task participants responded to a white circle with a button press and inhibited a prepotent response when a white diamond signaled to withhold the response. One of the fractal image cues was followed by a go signal in 80% of the trials and in 20% by a no-go signal (and vice versa for the second fractal cue). Cues were framed in one of the colors associated with feedback during training

*Within-subject variant of the cued go-/no-go task* Participants underwent the same training as in experiment 1. In the test phase of the experiment each participant performed a variant of the ABBA task, which is illustrated in Fig. 4. To be able to compare the effects of reward-associated stimuli on performance in the test phase within-subject, we modified the cues: each trial one of two fractal images was presented as a cue, predicting the probability of a go or a no-go response. One of the fractal images was followed by a go signal in 80% of the trials and by a no-go signal in 20% of the trials. For the other fractal image probabilities were reversed. The fractal images were framed in one of the colours associated with the experimental conditions to investigate the impact of reward history on inhibitory performance. As a minor change we used independent buttons for the training (arrow-left, arrow-right) and the test phase (arrow-upwards). As there were no interaction effects of the SOA condition in our previous data and for the sake of test duration cues were all presented for 300 ms. Like in the previous experiment participants performed 112 go-trials versus 28 no-go-trials per experimental condition, which resulted in a total number of 280 trials (with a duration of about 25 min).

*Power calculations for experiment 2* Post-hoc power calculations using GPower 3 (Faul et al., 2007) indicated that we would have found a significant effect of value in the test phase of the experiment (medium sized, *f* = 0.25) with a probability of α = 0.05 and actual power of β = 0.84 in a sample of this size (N = 71 participants), if there was any.

### Results of experiment 2

#### Training phase

Data pre-processing was equivalent to the procedure used for experiment 1. Average accuracy in the training phase was 90.14%. We removed 0.7% of the responses, because they were slower than three standard deviations above the mean, while no responses were faster than three standard deviations above the mean. As for experiment 1 we conducted Bayesian repeated-measures analysis of variance with the factors feedback type and block.

Like in experiment 1 participants responded faster when they were rewarded with money, compared to the neutral feedback condition. The Bayesian results convincingly questioned the predictive value of the null hypothesis, strongly supporting the existence of a value-driven response bias in the training phase (BF10 = 19.898). Responses became overall faster throughout the training (BF10 = 1.144e + 10). The frequentist analysis (compare supplementary material) suggested a steeper decline of response times in the reward condition, but the Bayesian analysis indicated that an interaction with feedback type was moderately unlikely for this data (BF-inclusion = 0.231).

**Fig. 5:**
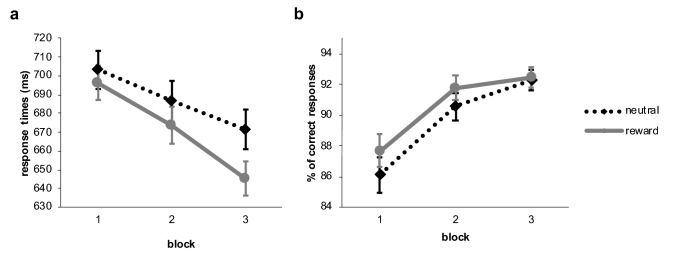
Response times of correct responses (**a**) and accuracy of responses (**b**) per block and feedback condition during training of experiment 2. Error bars reflect the standard error of means

Second, we analysed accuracy of responses in the training phase: our participants responded overall more accurate throughout the training phase (BF10 = 1.867e + 11). Regarding an influence of feedback type on accuracy of responses graphical exploration suggested participants responded more accurate in rewarded compared to neutral feedback trials, but this was statistically not significant (compare supplementary material) and the Bayesian analysis confirmed the data was three times more likely to occur under the null hypothesis (BF10 = 0.355). An interaction between feedback type and block was strongly unlikely to occur in the data (BF-inclusion = 0.082). Figure 5 illustrates the results for the training phase of experiment 2.

#### Test phase

Data cleaning was identical to the previous ones and resulted in removal of 0.8% of the responses during go-trials. Average accuracy in the test phase was 99.67% for go-trials and 89.13% for no-go trials. We conducted Bayesian repeated-measures analysis of variance using the within factors fractal cue (cue predictive of a go or no-go response) value (reward or neutral feedback) and block. Analyses were calculated separately for action (go) versus inaction (no-go) trials and for both, response times and accuracy measures, resulting in four analyses of variance (also see supplementary material).

**Fig. 6:**
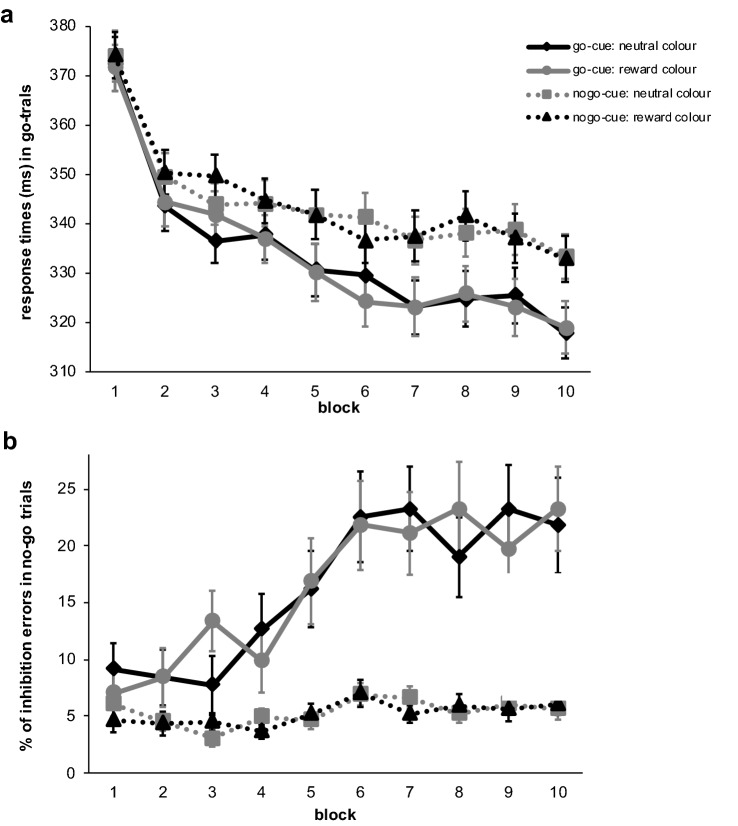
Results of the test phase of experiment 2: graph **a** shows response times in go-trials over the time course of the test phase. Graph **b** illustrates accuracy of responses in no-go trials, which was converted into percentage of inhibition errors for illustration purposes. Error bars reflect the standard error of means

*Accuracy in no-go trials:* First of all, our data analysis revealed that participants learned to associate the fractals with a certain response throughout the test phase (fractal: BF10 = 1.012e + 47, fractal*block: BF-inclusion = 87,582.538). The fractal cue mostly followed by a go signal, induced difficulties to inhibit responses, whereas inhibitory performance after cues associated with inaction remained relatively stable over the course of the test phase (see Fig. 6b). Correspondingly, results for factor block suggested subjects conducted overall more errors over the course of the test phase (BF10 = 1.196e + 9). But we found strong evidence against a value-driven disinhibitory effect. The Bayesian analysis for factor value indicated that the data was 24 times more likely to occur under the null hypothesis (BF10 = 0.042). The BFs for the interactions with block (BF-inclusion = 1.830e−4), with fractal cue (BF-inclusion = 0.060) as well as three-way (BF-inclusion = 2.945e−4) suggested strong to extreme evidence for the null hypothesis, clearly questioning the expected influence of the reward-associated cues on motor inhibition.

*Response times in go-trials:* Similarly, to the accuracy in no-go trials the Bayesian analysis for response times in go-trials indicated learning of the contingencies did take place (fractal: BF10 = 5.478e + 18), although for the response times the interaction between fractal cue and block was strongly predicted by the null hypothesis (BF-inclusion = 0.041). Results for factor block showed that responses became faster over the course of the test phase (BF10 = 1.276e + 129). Importantly, the Bayesian analysis revealed strong evidence against our expectations and in favour of the null hypothesis in case of factor value (BF10 = 0.044) as well as strong to extreme evidence regarding an interaction with fractal cue (BF-inclusion = 0.057), with block (BF-inclusion = 2.479e−4) and three-way (BF-inclusion = 1.331e−4) suggesting the data would likely occur under the null-hypothesis. View Fig. 6a for an illustration of these results.

*Discussion of experiment 2* Experiment 2 was performed to further examine the inconclusive results from experiment 1 by comparing value-driven effects on motor inhibition within-subject in another large sample. Like in experiment 1 statistical analysis showed that participants learned to associate colours with feedback during training. Again, the frequentist and Bayesian statistical approach were in line with each other and with our expectations. Furthermore, we were able to clearly demonstrate that participants learned to associate the fractal cues with action/inaction throughout the test phase and the data suggested stable motivation for the task. But we did not find any evidence that irrelevant stimuli which carry reward information interfere with motor inhibition in an independent subsequent go-/no-go task. The lack of a disinhibitory effect of the reward-associated features of the cues indicates that if there was any response activation triggered by them, our participants managed to overcome it to perform well in situations that require response inhibition. However, there was also no sign of stronger approach activation in trials containing formerly reward predictive cues. This lack of evidence for an influence of reward-history on subsequent action control stands in contrast to previous findings from studies that also used a separate training and test phase (e.g., Anderson et al., 2016; Kim & Anderson, 2019; van Wouwe et al., 2015).

## General discussion

The current study investigated if previously reward-predictive stimuli, which modulate attention and typically cause distraction in goal-directed visual search (e.g., Anderson & Halpern, 2017; Marchner & Preuschhof, 2018; Theeuwes, 2018), would promote response activation and handicap inhibition of motor responses in an unrelated task. In a number of experiments, it has been shown that reward-predictive stimuli can impede goal-directed actions and inhibitory control under conditions, where reward feedback is still available (e.g., Gluth et al., 2018; Krebs et al., 2013; Wang et al., 2018). Additionally, a few researchers have demonstrated that associative reward learning in one task can impede inhibitory processes in unrelated subsequent tasks, after removal of reward feedback, rendering the value-signalling stimuli irrelevant (Anderson et al., 2016; Kim & Anderson, 2019; van Wouwe et al., 2015). We aimed to extend this line of research by combining two classic paradigms. In two experiments participants first underwent a training phase, in which they learned to associate colours with a monetary or a neutral feedback, which typically biases attentional selection. Afterwards participants performed a cued go/no-go task that required fast responses in frequent go-trials and withholding a motor response in rare no-go trials. Importantly, the colour of the cue was associated with reward or neutral feedback in the learning phase. Considering previous research, we expected that the learned value linked to the cues would promote activation and interfere with inhibition of frequently executed responses, causing difficulties to flexibly control behaviour when approach is inadequate. Unexpectedly, we did not find convincing evidence for this hypothesis in the first experiment, while the second one, which was a replication of the first one using a within-subject design, clearly supported the null hypothesis indicating no effect of reward history on response inhibition. In the first experiment, when participants had to inhibit a prepotent action, error rates for cues associated with reward feedback were larger compared to error rates for cues associated with neutral feedback, while results from the Bayesian analysis were inconclusive. The second experiment clearly showed that error rates were not affected by reward history. Also, there was no sign of stronger approach tendencies as a reaction to value-associated cues as response times in go-trials did not vary as a function of value in both experiments, which questions that reward history influenced behavioural activation. The null-results we report here were validated using the Bayesian statistical approach, which suggested that effects of reward-associated stimuli on the performance in the test phase were likely under the null-hypothesis. Learned stimulus-reward associations, which have previously been shown to bias selective attention (see, e.g., Anderson & Halpern, 2017; Marchner & Preuschhof, 2018; Theeuwes, 2018), did not handicap the inhibition of responses.

Importantly, in the training phase of both experiments we observed a significant effect of reward on response speed. Reward feedback clearly affected behaviour as long as rewards were still available which suggests successful learning of the stimulus–outcome-associations. Additionally taking into account the numerous studies showing that this kind of training with a similar number of repetitions typically affects behavior in subsequent tasks and after removal of reward feedback, e.g., during visual search (Anderson et al., 2011, Anderson & Halpern, 2017, Marchner & Preuschhof, 2018, or see reviews by Anderson, 2013; Theeuwes, 2018), it seems unlikely that a potential deficiency of the training accounts for the null effects of reward history on motor inhibition in the test phase. In a recent study, generalization of stimulus-reward learning has even been demonstrated for semantic synonyms of words that were paired with reward using the exact same number of repetitions in the training phase and which later caused interference in a subsequent Stroop task (Liao et al., 2020). However, a replication failure regarding generalization of a value-driven response bias from the training to another task cannot be completely ruled out. To exclude this possibility, additional demonstration of, e.g., a typical value-driven attention capture effect would be insightful in future research. But this approach would risk promoting extinction, which is more likely to occur with more repetitions under reward omission and can in turn account for a lack of evidence as well (Roper et al., 2014). On the other hand, attentional biases have proven to be quite robust (Anderson et al, 2011; Jiang et al., 2013). Concerning possible extinction effects in our experiments it should be noted that we did not observe interactions between value and block in the test phases which speaks against this line of reasoning.

Taken together reward history did not impact behavioural activation or motor inhibition in a subsequent cued go/go-go task. This stands in contrast to previous studies addressing the relations between associative learning, selective attention and the executive control of action. Studies that directly investigated a link between previously reward-predictive stimuli and action activation have shown that reward-associated stimuli interfere with inhibitory processes (Anderson et al., 2016; Kim & Anderson, 2019; van Wouwe et al., 2015; Wang et al., 2018). As a theoretical model this interference has largely been interpreted as a direct influence of associative reward learning on behavioural activation (Anderson et al., 2016; Wang et al., 2018), which has then to be inhibited by neural control circuits (Freeman & Aron, 2016; Freeman et al., 2014; Wang et al., 2018). In our experiments activation and inhibition in a simple, classic motor inhibition task remained unaffected by stimuli that were reward-predictive in the past. Considering the evidence of the summarized research reward-associated stimuli can influence inhibitory processes. At the same time our results highlight that consequences like unbeneficial choices or actions do not necessarily emerge.

Strictly speaking our finding is also not a complete mismatch with other experiments using a separate training and test phase, because these experiments demonstrated an impact of reward-associated distractors in the form of response time interference effects but did not find any impact of reward-history on accuracy of responses (Anderson et al., 2016; Kim & Anderson, 2019; Wouwe et al., 2015). For example, Wouwe et al. (2015) observed no differential influence of value-action couplings on the percentage of impulsive errors. In contrast, slopes of reaction time interference effects revealed two findings: first, engagement of inhibitory control for the couplings ‘inaction for reward’ and ‘action to avoid punishment’, and second suppression of interference was disrupted in the more ‘natural’ couplings (action–reward, inaction–punishment). The 2-phasic experiments which manipulated stimulus–response compatibility found value-driven alterations of response conflicts only in indirect measures (e.g., slowing of response time). The classic go/no-go task design we used also measures response conflict resolution but is an estimate of a person’s accuracy to withhold a frequently executed, relatively automatic response in a very simple task. It is a direct measure of motor inhibition, because it estimates inhibition through behavioural errors not indirectly through response time interference. To measure the actual manifestation of errors seems more naturalistic as it indicates how strong reward-associated stimuli really impact behavioural choices. Our results suggest that this impact was not considerable, at least after this kind of training procedure. An advantage of indirect measures seems to be the sensitivity in detecting influences of stimuli with a history of reward. Humans have proven to be skilled in adjusting their behaviour flexibly in relatively simple laboratory tasks, but the resolution of cognitive conflict can still cost measurable response time. For example, lately a series of experiments showed that after overtraining stimulus–response–outcome associations, humans continue choosing correct actions when the outcome contingencies change (Luque et al., 2019). The experiments even suggested that with longer training reward-driven response selection errors tend to decrease, implying that actions become more goal-directed. Instead, the reaction time interference with ongoing goal-directed behaviour reliably indexed unfavourable habitual response patterns. Thus, even after extended periods of associative training, it has proven to be difficult to measure disadvantageous automatic actions in humans in laboratory task settings (de Wit et al., 2018; Luque et al., 2019; Watson & de Wit, 2018). Likely situational features (in this case features of the task design) as well as individual differences determine if reward history considerably influences processes of behavioural activation and inhibition which lead to unbeneficial actions. Studying the protective factors that allow adequate response selection in the face of misguiding irrelevant reward-associated stimuli would be interesting future research considering the implications for behavioural and substance addictions. In healthy populations failures in resisting tempting distractors were found to be related to neural responses in a salience detection network (Steimke et al., 2017) and areas for performance monitoring (Krönke et al., 2018). Such individual differences may in part constitute differences in the ability to overwrite response tendencies triggered by value-signaling stimuli.

Related research about valence-action biases suggests that the impact of irrelevant reward-associated stimuli also depends on the awareness of the contingencies (Failing & Theeuwes, 2017; Liao et al., 2020) and on whether they are perceived before or simultaneously with the response signal (Hoofs et al., 2019). Hoofs et al. (2019) reported that both, reward- and punishment-associated targets facilitated approach but impaired avoidance, while valence-related cues, had a generally positive effect on performance. Thus, the time of occurrence of the valanced stimulus (before or together with the response signal) seems to modify its effects. In the test phases of our experiments reward-associated stimuli were always presented before the target, as a cue or a cue feature, which enhanced overall performance in Hoofs et al.’s (2019) study and had no effect in ours. Also, informing participants about an upcoming reward-associated distractor during goal-directed search did reduce distraction by reward history enhancing goal-directed control in another study (Failing & Theeuwes, 2017). Therefore, leaving time between a value-associated distractor and the moment of action can be beneficial for behavioural control. In our experiments only the cues were associated with value, leaving some time for goal-directed and less automatic processing. This aspect may have contributed to behavioural control in our study, and, therefore, may have complicated the demonstration of a value-driven disinhibition.

On the other hand, experiments using very similar paradigms, like variants of the go/no-go task with emotional cues (e.g., facial or otherwise valanced pictorial stimuli), show clearly that such stimuli promote approach behaviour accompanied by inhibitory errors (Hare et al., 2005; Schulz et al., 2017). Also, when substance-related images are presented as go-cues response inhibition is impaired in patients with substance use disorder (Pike et al., 2015; Weafer & Fillmore, 2012, 2015). Compared to these cues, the training of colour-reward cues in our experiments was a lot less intense, less generalised and we used secondary rewards as reinforcers. Therefore, emotional or addiction-related cues are likely to be much more salient compared to the reward-associated cues we used which were learned by associating an originally neutral colour and a monetary or a neutral feedback. Studies using motivational or substance-related cues in go/no-go tasks teach that such valanced stimuli do interfere with motor inhibition and suggest that incentive salience is crucial for these effects (Dill & Holton, 2014; Robinson & Berridge, 2008). In the contrary our experiments showed that value-associated stimuli do not necessarily impact inhibitory performance when these stimuli are task-irrelevant and of lower salience. It can be inferred that while attentional biases seem to be learned fast and effortless and tend to be robust and inflexible (Jiang et al., 2013; Theeuwes, 2018) a considerable impact of irrelevant reward-associated stimuli on the ability to inhibit unfavorable actions may evolve slower, with more repetitions or stronger incentives. Our finding points to resources that are available for the compensation of response biases induced by stimuli with a history of reward.

In the current study we aimed to expand the perspective on the relations between reward learning, selective attention and action formation. In summary, our findings imply that now irrelevant, but reward-signalling stimuli do not necessarily have an impact on subsequent actions and inhibitory performance. We did not observe inhibitory deficits in the face of previously valuable stimuli in a motor inhibition task. This highlights compensatory resources available to overcome influences from Pavlovian learning. The mismatch with previous findings could be due to variations in task design and the motivational salience of the stimuli supporting that certain task and stimulus characteristics can promote and, more importantly, can decrease unbeneficial action tendencies driven by value. Studying circumstances which help to overcome disinhibition triggered by reward-associated stimuli seems an interesting field for future investigations.

## Supplementary Information

Below is the link to the electronic supplementary material.

Supplementary file1 (PDF 136 KB)
