# Supplementary material for: The influence of associative reward learning on motor inhibition
Source: Psychol Res. 2021 Feb 17;86(1):125–40. doi: 10.1007/s00426-021-01485-7 (PMC8821474; doi:10.1007/s00426-021-01485-7)
Supplement: Supplementary file 1 — Supplementary file1 (PDF 136 KB) [file 426_2021_1485_MOESM1_ESM.pdf]

Supplementary material

Article: The influence of associative reward learning on motor inhibition.

Authors: Janina Rebecca Marchner [1,2] & Claudia Preuschhof [1,2]

Affiliations: [1] Department of Clinical Developmental Psychology, Otto-von-Guericke University, 39106 Magdeburg, Germany, [2] Center for Behavioral Brain Sciences, 39106 Magdeburg, Germany

Journal: Psychological Research

Correspondence contact: claudia.preuschhof@ovgu.de

Additional results

Statistical frequentist analyses were carried out using SPSS (IBM Corp., 2013) and Bayesian statistical analyses were carried out using JASP (JASP Team, 2020, Version 0.14) with standard settings for priors. Correct response time and accuracy were analysed using repeated measures analysis of variance (ANOVA). The analyses for the training phases were conducted using the within subject factors feedback type (reward, neutral) and block. The test phase of experiment 1 was analysed using SOA (100 ms versus 300 ms) and block as within-subject factors and experimental group as between subject factor, which reflected the trained associations with reward versus neutral feedback. For the test phase of experiment 2 the impact of reward associations was analysed using the within-subject factor 'value' (reward versus neutral); the within-subject factor 'fractal cue' reflected the associated response (go versus no-go) and the within-subject factor block was also integrated in the model. The Bonferroni method was used for post-hoc correction of multiple comparisons. Violation of the sphericity was corrected using Greenhouse-Geisser method. For task measures not described in the main article, results of both, the Bayesian analysis using JASP and analysis of variance using SPSS are presented.

Experiment 1, training phase:

Analysis of variance using SPSS revealed a significant main effect of feedback type on response times (F(1, 75) = 16.20, $p < .001$, $\eta_p^2 = .18$) indicating that responses were faster during rewarded trials. Responses also became faster over the time course of the experiment indicated by a main effect of experimental block (F(2, 150) = 41.38, $p < .001$, $\eta_p^2 = .36$) and by the significant post-hoc comparisons (all comparisons: $p < .001$). Block interacted with feedback type (F(2, 150) = 13.29, $p < .001$, $\eta_p^2 = .15$) indicating a steeper increase of response speed in rewarded trials compared to unrewarded feedback trials. Reaction times of neutral and rewarded trials differed from block to block as indicated by post-hoc repeated contrasts [reward<neutral – block1 vs. 2: F(1, 75) = 05.28, $p < .05$, $\eta_p^2 = .07$; block2 vs. 3: F(1, 75) = 09.80, $p < .01$, $\eta_p^2 = .12$].

The same repeated-measures ANOVAs were calculated for accuracy of responses. A significant effect for the factor block showed that performance improved overall throughout the time course of the training phase (F(1.68, 126.00) = 28.20, $p < .001$, $\eta_p^2 = .27$; Greenhouse-Geisser corrected). Post-hoc pairwise comparisons specified this improvement stagnated after the second block (block1 vs. block2: $p < .001$; block1 vs. block3: $p < .001$; block2 vs. block3: $p > .1$). Accuracy of responses was higher in rewarded compared to unrewarded trials, but this difference was not significant (F(1, 75) = 2.31, $p > .1$). The interaction between block and feedback type revealed also no differences between the conditions over time (F(2, 150) = 0.57, $p > .1$).

Experiment 1, test phase:

Accuracy in no-go trials following (invalid) go-cues: Analysis of variance using SPSS revealed that accuracy was significantly lower in the group that associated the cue with reward (main

factor experimental group: F(1, 74) = 4.01, $p < .05$ $\eta_p^2 = .05$). Factor SOA reached significance (F(1, 74) = 8.85, $p < .01$, $\eta_p^2 = .11$) suggesting that overall less inhibitory failures were made when responses were given after 100 ms compared to 300 ms. As experience with the probabilities proceeded over blocks, participants committed more impulsive errors (block: F(4.76, 352.24) =, $p < .1$, $\eta_p^2 = .03$, Greenhouse-Geisser corrected), a trend which suggests the development of automation of the frequent response. Block did not interact with other factors ($p > .1$).

Reaction times in go-trials following (valid) go-cues: The factor experimental group failed to reach statistical significance in the frequency analysis (F(1, 74) = 2.62, $p = .110$) and did not interact with block (F(6, 432) = 0.12, $p > .1$). Responses became overall faster over the course of the test phase as indicated by a significant main effect for the factor block (F(3.86, 285.90) = 10.72, $p < .001$, $\eta_p^2 = .13$, Greenhouse-Geisser corrected). We found a significant main effect of the factor SOA on response times (F(1, 74) = 261.67, $p < .001$, $\eta_p^2 = .78$) but factor SOA did not interact with factor experimental group (F(1, 74) = 2.97, $p = .09$, $\eta_p^2 = .04$). All other interactions were also not significant ($p > .1$).

In addition to these two conditions of interest we also analysed the following data: *Accuracy in no-go trials following (valid) no-go cues:* When participants had to remain inactive after cues that were mostly followed by a no-go signal, an influence of value on accuracy should manifest as higher accuracy in trials in which the cue was associated with a neutral feedback versus monetary reward. We observed no differences between the experimental groups (F(1, 74) = .13, $p > .1$) and no interactions with the within subject factors ($p > .1$). The Bayesian analysis indicated that the hypothesis of a value-driven group difference in this variable was six times more likely under the null hypothesis (BF10 = 0.159). Like for response times in go-trials following no-go cues the results suggested that the learned associations with value had no influence when cues were associated with inaction. Responses became overall more accurate over the course of the test phase (block: F(5.05, 373.41) = 5.8, $p < .001$, $\eta_p^2 = .07$, Greenhouse-

Geisser corrected; BF10 = 720.520). For the factor SOA the data analysis was overall not informative (SOA: $F(1, 74) = 3.32$, $p < .1$, $\eta_p^2 = .04$; BF10 = 1.015). All other factors did not reach statistical significance and did not interact with each other in the frequentist analysis ($p > .1$). The Bayesian factors regarding the interactions between experimental group and block (BF-inclusion = 0.006) as well as the three-way interaction (BF-inclusion = 0.025) were in favour of the null hypothesis. For the interaction between experimental group and SOA we did not find informative results (BF-inclusion = 0.253)

Reaction times in go-trials following (invalid) no-go cues: When participants had to give a response after cues that were frequently followed by a no-go signal, we would expect to observe faster responses in the reward versus neutral experimental condition, because the association with reward is expected to activate a response and thus should help to overcome the conflict between the cue and the response signal. But we observed no difference between the experimental groups in response times after no-go cues ($F(1, 74) = .00$, $p > .1$). An influence of value on response times was moderately unlikely predictive of this data (BF10 = 0.194). The interactions of experimental group with SOA ($F(1, 74) = .06$, $p > .1$; BF-inclusion = 0.134) or block ($F(1, 74) = .57$, $p > .1$; BF-inclusion = 0.003) and the three-way interaction ($F(6, 444) = .21$, $p > .1$; BF-inclusion = 0.012) were also not significant and were likely under the null hypothesis according to the Bayesian analysis. Responses to go targets following no-go cues were slower in the short compared to the long SOA ($F(1, 74) = 42.20$, $p < .001$, $\eta_p^2 = .37$), which was validated by the Bayesian analysis with extreme evidence (BF10 = 7.087e+11). A significant effect for block on response times (F($F(4.90, 362.78) = 3.15$, $p < .01$, $\eta_p^2 = .04$, Greenhouse-Geisser corrected) indicated that response times became shorter throughout the test phase, but the Bayesian factor was not very informative in this case with a BF close to zero (BF10 = 0.480). Block interacted with SOA ($F(4.96, 368.17) = 2.49$, $p < .05$, $\eta_p^2 = .03$, Greenhouse-Geisser corrected; BF-inclusion = 0.054), showing that the difference between the

SOA conditions became smaller throughout the course of the test phase. None of the repeated contrasts of the interaction reached significance.

Accuracy in go-trials following (valid) go-cues: Analysis of variance using SPSS revealed that participants responded more accurate in go-trials in the group that associated the cue with reward, but this difference did not reach significance (main factor experimental group: F(1, 74) = 2.98, $p < .1$, $\eta_p^2 = .04$) and the Bayesian analysis revealed moderate evidence for the null hypothesis (BF10 = 0.224). Factor SOA (F(1, 74) = 1.64, $p > .1$; BF10 = 0.172) and block (F(4.58, 339.211) = 0.74, $p > .1$, Greenhouse-Geisser corrected; BF10 = 0.002) were also non-significant and BFs supported the assumption of null hypothesis. All interactions were non-significant ($p > .1$) and results were clearly in favour of the null hypothesis (soa*block: BF-inclusion = 0.005; experimental group*soa: BF-inclusion =0.146; experimental group*block: BF-inclusion =0.005; experimental group*block*soa: BF-inclusion =0.075).

Accuracy in go-trials following (invalid) no-go cues: When participants had to press a button after a conflicting cue there was no reward-associated difference as indicated by the missing group effect (factor experimental group: F(1, 74) = 0.00, $p > .1$), which was 10 times more likely to be found under the null hypothesis according to the Bayesian approach (BF10 = 0.095). Factor SOA (F(1, 74) = 1.15, $p > .1$; BF10 = 0.143) and block (F(4.37, 323.41) = 0.74, $p > .1$, Greenhouse-Geisser corrected; BF10 = 0.001) did not reach significance like all interactions ($p > .1$). BFs for the interactions suggested extreme evidence in favour of the null hypothesis (soa*block: BF-inclusion = 2.448e-5; experimental group*soa: BF-inclusion =0.004; experimental group*block: BF-inclusion = 1.522e-6; experimental group*block*soa: BF-inclusion = 3.393e-10).

Response times in no-go trails: Both models for trials containing valid and invalid cues could not be estimated, because there were not enough cases.

Experiment 2, training phase:

Analysis of variance using SPSS revealed a significant effect of feedback type on response times with faster responses in rewarded trials (F(1, 70) = 4.59, $p < .05$, $\eta_p^2 = .06$) and feedback type interacted with block (F(1.83, 128.25) = 5.44, $p < .01$, $\eta_p^2 = .07$, Greenhouse-Geisser corrected) indicating steeper decline of response times in the reward condition. Responses became faster with more training as indicated by a significant effect of block (F(1.81, 126.33) = 41.84, $p < .001$, $\eta_p^2 = .37$, Greenhouse-Geisser corrected; with all post-hoc comparisons $p < .001$).

Further, analysis of variance suggested that participants responded overall more accurate over the course of the training phase (F(1.59, 111.25) = 30.02, $p < .001$, $\eta_p^2 = .30$, Greenhouse-Geisser corrected). There was no statistically significant effect of feedback type on accuracy of responses (F(1, 70) = 2.22, $p > .1$) and we did not find an interaction with block (F(2, 140) = 0.76, $p > .1$).

Experiment 2, test phase:

Accuracy in no-go trials: Like the Bayesian analysis, the analysis of variance using SPSS also supported that the go/no-go paradigm worked as intended, as factor fractal cue (F(1, 75) = 76.46, $p < .001$, $\eta_p^2 = .51$) and the interaction with block (F(6.479, 485.95) = 9.23, $p < .001$, $\eta_p^2 = .11$, Greenhouse-Geisser corrected) were highly significant. Overall subjects conducted more errors over the course of the test phase (F(6.87, 515.15) = 7.22, $p < .001$, $\eta_p^2 = .08$, Greenhouse-Geisser corrected). But the associations with value linked to the colour surrounding the cue did not impact inhibitory performance as indicated by the results for factor value (F(1, 75) = 0.02, $p > .1$), the interaction with block (F(7.49, 562.41) = 0.75, $p > .1$, Greenhouse-Geisser

corrected), the interaction with fractal cue ($F(1, 75) = 0.05$, $p > .1$) and the three-way interaction ($F(7.41, 555.98) = 0.59$, $p > .1$, Greenhouse-Geisser corrected).

Response times in go-trials: Further, the analysis of variance suggested that learning of the contingencies was sufficient in the test phase as indicated by significant results for factor fractal cue ($F(1, 75) = 124.48$, $p < .001$, $\eta_p^2 = .66$) and the interaction with block ($F(6.45, 483.77) = 24.10$, $p < .001$, $\eta_p^2 = .24$, Greenhouse-Geisser corrected). Overall responses became faster over the course of the test phase ($F(5.50, 412.60) = 30.79$, $p < .001$, $\eta_p^2 = .29$, Greenhouse-Geisser corrected). We did not observe stronger approach behaviour in trials containing a previously reward predictive cue: results for factor value ($F(1, 75) = 0.26$, $p > .1$), the interaction with block ($F(7.41, 555.57) = 0.90$, $p > .1$, Greenhouse-Geisser corrected), the interaction with fractal cue ($F(1, 75) = 1.20$, $p > .1$) and three-way interaction ($F(9, 657) = 0.50$, $p > .1$) were all non-significant.

Accuracy in go trials: The Bayesian approach did not yield informative results concerning an effect of value in go trials (value: BF10 = 1.976), but there was moderate to extreme evidence for the null hypothesis looking at the interaction terms (value*block: BF-inclusion =0.127; fractal*value: BF10 = 0.058; fractal*value*block: BF-inclusion = 1.716e-4). The other factors (fractal: BF10 = 0.048; block: BF10 = 8.182e-5) and the interaction (fractal*block: BF-inclusion = 1.879e-5) were also clearly predicted by the null hypothesis. The analysis of variance using SPSS suggested there was no difference in accuracy depending on whether the cue predicted a go or no-go response (fractal: $F(1, 75) = 3.37$, $p < .1$, $\eta_p^2 = .04$; fractal*block: $F(5.88, 6.160e-5) = 0.54$, $p > .1$, Greenhouse-Geisser corrected). Trend-wise accuracy was higher in the reward condition (value: $F(1, 75) = 3.95$, $p = .51$, $\eta_p^2 = .05$). When participants had to give an active response by pressing a button and the cue promoted this action, subjects responded more accurately when the cue's outline colour additionally was linked to reward. However, this did not reach significance and only very few behavioural failures constitute these results because accuracy in go trials was overall very high (valid: $M =$

99.68 , *SD* = 0.01, invalid: *M* = 99.65 , *SD* = 0.01). Block did not interact with value (F(7.19, 538.84) = 0.91, *p* > .1, Greenhouse-Geisser corrected), nor with value and fractal-cue (F(1, 75) = 0.21, *p* > .1) and block itself was also not significant (F(6.23, 467.21) = 1.42, *p* > .1, Greenhouse-Geisser corrected).

Response times in no-go trails: could not be estimated, because there were not enough cases.
